# Fabrication and Characterization of Solution Blow Spun Zinc Oxide Nanoparticles/Polyvinyl Butyral Nanofiber Membranes for Food Packaging

**DOI:** 10.3390/polym18020195

**Published:** 2026-01-10

**Authors:** Mengyu Zhang, Wenqian Han, Mingfu Zhang, Yesheng Zhong, Liping Shi, Xi Chen

**Affiliations:** 1 National Key Laboratory of Science and Technology on Advanced Composites in Special Environments, Harbin Institute of Technology, Harbin 150080, China; 2 The Second Affiliated Hospital of Harbin Medical University, Harbin 150006, China

**Keywords:** PVB, solution blow spinning, nanofiber membrane, ZnO NPs, antibacterial, food packaging

## Abstract

To address the low-value recycling dilemma of waste polyvinyl butyral (PVB) and cater to the demand for sustainable multifunctional active food packaging, this study developed a facile and cost-effective solution blow spinning approach. Continuous, smooth, and bead-free nanofiber membranes were prepared by optimizing the solution blow spinning process parameters. Zinc oxide nanoparticles (ZnO NPs) were incorporated into the PVB nanofiber membrane with vacuum impregnation. The results demonstrated that ZnO NPs significantly enhanced the tensile strength, thermal stability, and the UV absorption of PVB fiber membranes. ZnO/PVB fiber membranes exhibited antibacterial activity against *Staphylococcus aureus*, *Escherichia coli*, and *Pseudomonas aeruginosa*. Practical preservation tests showed that ZnO/PVB fiber membranes effectively inhibited cherry tomatoes’ microbial spoilage and water loss, extending the shelf life of tomatoes to 13 days. These findings validate the potential of ZnO/PVB composite nanofiber membranes as active food packaging and provide a feasible technical pathway for the low-cost, efficient utilization of recycled PVB.

## 1. Introduction

Food packaging serves as a critical link between food production and consumption, playing a central role in ensuring food quality and safety throughout the supply chain [1,2]. Inadequate packaging performance during transportation and storage continues to result in significant food waste annually due to spoilage and deterioration [3,4]. An ideal food package requires the following key properties: excellent barrier properties against oxygen, water vapor, oils, and flavor compounds to delay oxidation, moisture migration, and flavor loss; sufficient mechanical properties, such as tensile strength, elongation at break, and flexibility, to maintain integrity throughout logistics; and finally, reliable safety compliance with food contact standards along with good heat sealability, with both being prerequisites for commercial application [5,6]. Additionally, to address specific requirements, food packaging may also be engineered with functionalities like antibacterial properties, UV resistance, and controlled optical transparency in order to achieve the goal of prolonging shelf life and reducing food loss [7,8].

PVB is a non-toxic polymer, renowned for its excellent film-forming properties, physicochemical stability, and mechanical strength, finding extensive applications in the construction, automotive, and photovoltaic industries for several decades [9,10,11]. Due to the lack of low-cost recycling technologies and the degradation of polymer properties during the reprocessing of waste PVB, PVB is often discarded during the recycling process, resulting in low utilization [12,13]. Research indicates that recycled PVB can be utilized in lithium-ion batteries, but its consumption in the lithium battery sector is limited [14]. Studies have shown that recycled PVB can be used as a substrate for antibacterial nanofiber membranes in filtration [15,16].

## 2. Materials and Methods

Methods for preparing polymer fiber membranes mainly include melt blowing, electrospinning, solution blow spinning, etc. Melt blowing can achieve efficient and continuous large-scale production and has been widely applied in fields such as filtration and adsorption materials, but the prepared fibers are mostly at the micrometer level [17,18]. Solution blow spinning is a low-cost, efficient, safe, and straightforward way to prepare nanofiber membranes and offers a new pathway for consuming recycled PVB [19,20]. PVB can be processed into nanofiber membranes through a spinning process [21,22].

However, neat PVB fiber membranes lack inherent antibacterial properties [23]. Research indicates that incorporating antibacterial nanoparticles into fiber membranes can enhance their antimicrobial properties [24,25]. ZnO NPs are commonly added in fiber membranes to increase antibacterial properties. ZnO NPs are non-toxic inorganic nanomaterials with excellent thermal stability and UV absorption capabilities [26,27]. The antibacterial mechanism of ZnO NPs is releasing zinc ions (Zn^2+^) and generating reactive oxygen species (ROS) [28]. Research indicates that parameters of solution blow spinning, such as air pressure, solution flow rate, needle inner diameter, and solution concentration, significantly influence fiber morphology and consequently affect the properties of fiber membranes [29].

This study pioneers a low-cost, high-efficiency resource utilization pathway to address the challenge of recycled PVB, by optimizing solution blow spinning parameters to produce smooth, continuous, bead-free PVB fiber membrane, and subsequently coating PVB membranes with ZnO NPs to enhance the antibacterial properties, thermal stability, mechanical properties, and UV protection. We successfully developed antibacterial fiber membranes featuring outstanding UV absorption and adequate tensile strength for food packaging. This approach not only provides a novel method for efficiently utilizing recycled PVB but also establishes a robust scientific foundation for its industrial applications in biomedicine and health protection.

### 2.1. Materials

Polyvinyl butyral (PVB, molecular weight = 120,000–150,000 g/mol) powder and Zinc oxide nanoparticles (ZnO NPs, average particle size 20 nm, purity ≥ 99.99%) were purchased from Shanghai Aladdin Biochemical Technology Co., Ltd. (Shanghai, China). Anhydrous ethanol was purchased as a solvent from Fuyu Chemical Co., Ltd. (Tianjin, China). Deionized water (DI) was prepared in-house. All materials were used directly without further purification.

### 2.2. Preparation of Spinning Solution

PVB powder was dissolved in ethanol and stirred for 5 h at 40 °C, forming 2 wt.%, 5 wt.%, and 8 wt.% PVB spinning solution. The solution was cooled to room temperature for the later spinning process.

### 2.3. Preparation of PVB and ZnO NPs/PVB Composite Nanofiber Membranes

**Figure 1:**
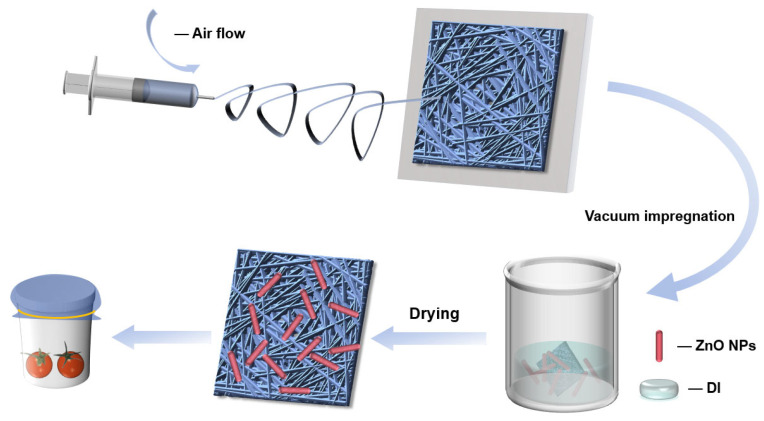
Schematic diagram of the preparation process of PVB and ZnO/PVB fiber membranes.

Figure 1 schematically illustrates the preparation process of PVB and ZnO/PVB fiber membranes. The spinning solution was loaded into a 10 mL syringe, which was then mounted onto a syringe pump. The needle tip was aligned to be level with and parallel to the collector, with a fixed collection distance of 15 cm. Airflow spinning was performed by controlling the air pressure (0.05–0.20 MPa), solution flow rate (0.05–0.20 mL/h), needle inner diameter (0.24–0.34 mm), and solution concentration (2 wt.%–8 wt.%). During the spinning process, the fibrous membranes were collected on a rotating drum. Then the fiber membranes were dried in an oven at 60 °C for 12 h to remove the solvent.

ZnO NPs were dispersed in DI and subjected to ultrasonic treatment (power: 300 W, duration: 30 min) to ensure uniform dispersion. The PVB fiber membrane was completely immersed in the ZnO NP dispersion and vacuum impregnated for 30 min at 50 mbar [30,31]. Then, the fiber membranes were dried at 60 °C for 6 h. In this study, ZnO NPs were added to PVB fiber membranes at concentrations of 3 wt.%, 5 wt.%, and 7 wt.% [32,33]. These PVB fiber membranes with ZnO NPs were designated as ZnO/PVB(X) (X represents the mass ratio of ZnO NPs).

### 2.4. Characterization

The morphology and elemental distribution of ZnO NPs and fiber membranes were observed using a field emission scanning electron microscope (FE-SEM, TESCAN MAGNA, Brno, Czech Republic) equipped with energy-dispersive spectroscopy (EDS). Samples were sputter-coated with gold (sputtering time: 210 s) prior to testing to enhance conductivity. Observations were conducted at an acceleration voltage of 5 kV. ImageJ software (Version 1.53 t) was used to analyze the average diameter of fibers. Twenty-five fibers were selected, and two points along each fiber were measured at equal intervals along the fiber’s longest axis. The diameter distribution reflects the fiber diameter range and average fiber diameter. The standard error indicates the uniformity of fiber diameter.

The water contact angle (WCA) of the fiber membranes was measured at room temperature using the static water drop method with an optical contact angle analyzer (JC2000D, Shanghai, China). A 0.5 μL drop of DI was placed on the surface of the fibrous membranes. Images of the water drop on the fiber membrane were recorded from 1 s to 60 s. WCA was calculated based on these images. To ensure measurement accuracy, three distinct locations were selected for testing on each sample.

The viscosity of PVB spinning solutions were measured at 25.0 ± 0.2 °C and 79.2 s^−1^ shear rate using a rotational viscometer (NDJ-5S, Shanghai, China).

The chemical functional groups and phase composition within the fiber membranes were analyzed at room temperature using a Fourier Transform Infrared Spectrometer (INVENIO S, Bruker, Germany). Spectra were collected over a wavenumber range of 4000 cm^−1^ to 400 cm^−1^.

The optical properties of fiber membranes were evaluated with Ultraviolet Visible (UV–Vis) spectroscopy, using a UV-Vis-NIR spectrophotometer (Cary 5000, Agilent Technologies Inc., Santa Clara, CA, USA). The spectroscopy was performed in a wavelength range of 200–800 nm.

The tensile strength of fiber membranes (PVB fiber membranes obtained through optimal preparation process parameters and PVB fiber membranes incorporating ZnO NPs with different mass ratios) was determined using a universal testing machine (UTM, SHK-A101, Suzhou, China). Fiber membranes were cut into strips (150 × 10 mm) for uniaxial tensile testing, with a crosshead speed at 10 mm/min. At least five replicate samples were tested for each specimen, and the average value was reported.

The thermal stability of the fiber membranes was evaluated using a thermogravimetric analyzer (TG, model Q50, TA Instruments, New Castle, DE, USA). Testing was conducted in a nitrogen atmosphere at a heating rate of 10 °C/min over the temperature range of 35 °C to 700 °C. The initial decomposition temperature of fiber membranes defined by a 5% TG mass loss (T_5%_) is a key thermal stability indicator

The antibacterial activity of the fiber membranes was evaluated using the agar diffusion method (Kirby–Bauer test). Tested strains included Staphylococcus aureus (*S. aureus*, ATCC 6538), Escherichia coli (*E. coli*, ATCC 25922), and Pseudomonas aeruginosa (*P. aeruginosa*, ATCC 27853). A single bacterial colony was inoculated into a Luria–Bertani (LB) broth and cultured until the optical density at 595 nm (OD_595_) reached approximately 0.3. The bacterial suspension was then diluted 10-fold in fresh LB broth to a concentration of approximately 10^7^ CFU/mL. A total of 100 µL of the diluted suspension (containing approximately 10^6^ CFU) was taken and spread evenly onto solid LB agar plates using a sterile spreader. Fiber membranes were cut into 10 mm diameter disks, sterilized by UV irradiation for 30 min, and then placed onto the agar surface under aseptic conditions. The plates were incubated at 37° C for 24 h, then observed, and the diameter of the inhibition zones was measured.

To evaluate the practical application of PVB fiber membranes in antibacterial fruit packaging, fresh cherry tomatoes (Lycopersicon esculentum var. cerasiforme A. Gray) were carefully selected from the market as research subjects, ensuring each tomato was comparable in size, color, and ripeness. For each experimental group, cherry tomatoes were placed in disposable paper cups, which were then sealed at the rim with fiber membranes. The experiment involved four test materials: PVB, ZnO/PVB(3) nanofiber membranes, ZnO/PVB(5) nanofiber membranes, and ZnO/PVB(7) fiber membranes. Tomatoes without fiber membrane coverage served as the blank control group. Throughout the experiment, direct contact between the fiber membranes and tomatoes was avoided. Experimental conditions were maintained at 25 °C and 50% relative humidity. During the 13 days of experiment, photographs were taken, and tomato weights were measured every 3 days to assess tomato changes and the efficacy of the antibacterial fiber membranes. Tomato weight loss was defined as the difference between initial weight and daily weights, with the percentage weight loss relative to initial weight calculated using the following formula:

$$\mathrm{Weight}\;\mathrm{loss}\;\mathrm{rate}=(M_{0}-M_{d})/M_{0}\times 100\%$$

where M_0_ is the initial weight of cherry tomatoes and M_d_ is the weight of the tomatoes after d days.

## 3. Results and Discussions

### 3.1. Effect of Air Pressure on Fiber Morphology

**Figure 2:**
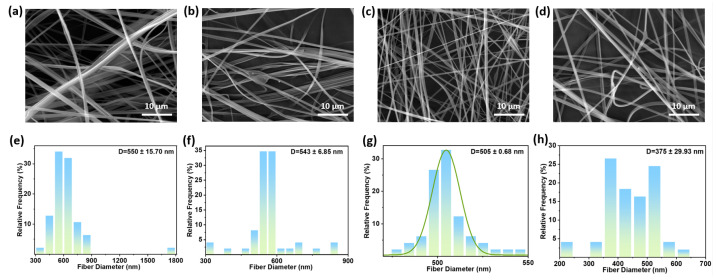
SEM images of PVB nanofiber prepared under air pressures of (**a**) 0.05 MPa; (**b**) 0.10 MPa; (**c**) 0.15 MPa; and (**d**) 0.20 MPa. Fiber diameter distribution of PVB nanofiber prepared under (**e**) 0.05 MPa; (**f**) 0.10 MPa; (**g**) 0.15 MPa; and (**h**) 0.20 MPa.

Air pressure is a critical parameter in solution blow spinning, as it determines the pneumatic tensile force acting on the polymer jet [34]. Figure 2 shows SEM images and fiber diameter distributions of fiber membranes prepared under different air pressures (solution flow rate 0.15 mL/min, nozzle inner diameter 0.26 mm, solution concentration 5 wt.%, collector distance 15 cm, room temperature). Under air pressure conditions of 0.05 MPa, Figure 2a,e show an average fiber diameter of 550 ± 15.70 nm. Insufficient pneumatic tensile force prevents the polymer jet from undergoing adequate deformation and refinement, resulting in larger fiber diameters and inter-fiber adhesion. When air pressure increases to 0.10 MPa, though beaded structures persist, the average fiber diameter is 543 ± 6.85 nm. Increased pressure reduces fiber diameter, but the polymer jet tensile force remains insufficient, resulting in beading within the fiber. At an air pressure of 0.15 MPa, the average fiber diameter is 505 ± 0.68 nm. Figure 2c reveals smooth, homogeneous, continuous, and bead-free fibers. This indicates an optimal balance between pneumatic tensile force and solution viscoelasticity, enabling stable Taylor cone formation and jet stretching. When air pressure increases to 0.20 MPa, the average fiber diameter is 375 ± 29.93 nm. Figure 2d reveals that excessive pneumatic tensile force caused over-stretching and instability of the jet stream, leading to morphological defects such as fiber splattering and breakage, which compromises the integrity of the fiber membranes.

### 3.2. Effect of Solution Flow Rate on Fiber Morphology

**Figure 3:**
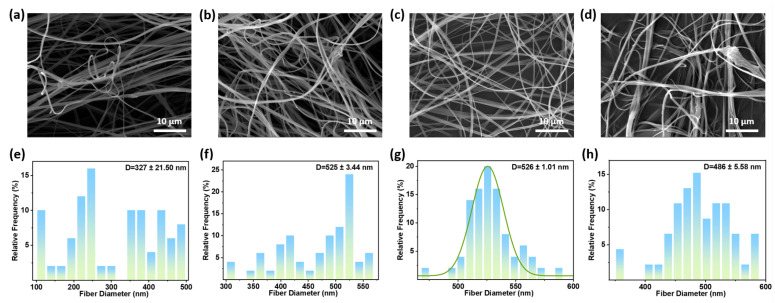
SEM images of PVB nanofiber prepared at solution concentrations of (**a**) 0.05 mL/min; (**b**) 0.10 mL/min; (**c**) 0.15 mL/min; and (**d**) 0.20 mL/min. Fiber diameter distribution of PVB nanofiber prepared at (**e**) 0.05 mL/min; (**f**) 0.10 mL/min; (**g**) 0.15 mL/min; and (**h**) 0.20 mL/min.

The solution flow rate directly determines the mass supply for polymer fiber formation [35]. Figure 3 shows SEM images and fiber diameter distributions of fiber membranes prepared under different solution flow rates (air pressure 0.15 MPa, nozzle inner diameter 0.26 mm, solution concentration 5 wt.%, collector distance 15 cm, room temperature). At a solution flow rate of 0.05 mL/min, the average fiber diameter is 327 ± 21.50 nm. When the solution flow rate increased to 0.1 mL/min, the average fiber diameter was 525 ± 3.44 nm. Under low flow rates, the solution could not maintain a constant air pressure. Low solution flow rates result in insufficient polymer solution supply. At constant air pressure, this causes excessive jet dilution, leading to jet breakage and the formation of discontinuous, smaller-diameter fibers. At a solution flow rate of 0.15 mL/min, the average fiber diameter is 525 ± 1.01 nm. The solution supply reaches a stable equilibrium with the pneumatic stretching and solvent evaporation rates, producing continuous, smooth, and uniformly distributed fibers. When solution flow rate increases to 0.20 mL/min (Figure 3d), the average fiber diameter is 486 ± 5.58 nm. Excessive solution delivery will impede the complete evaporation of the solvent, causing the fibers to stick together and form beads before reaching the collector.

### 3.3. Effect of Needle Inner Diameter on Fiber Morphology

**Figure 4:**
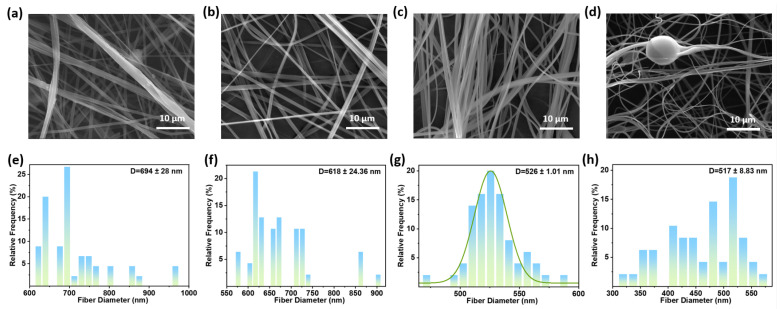
SEM images of PVB nanofiber prepared with a needle inner diameter of (**a**) 0.33 mm; (**b**) 0.31 mm; (**c**) 0.26 mm; and (**d**) 0.24 mm. Nanofiber diameter distribution of PVB nanofiber prepared with needle inner diameter of (**e**) 0.33 mm; (**f**) 0.31 mm; (**g**) 0.26 mm; and (**h**) 0.24 mm.

The needle inner diameter determines the initial diameter and stability of the polymer jet [36]. Figure 4 presents SEM images and diameter distributions of fiber membranes prepared using needles with different inner diameters (air pressure 0.15 MPa, solution flow rate 0.15 mL/min, solution concentration 5 wt.%, collector distance 15 cm, room temperature). With a nozzle inner diameter of 0.34 mm, the fibers have an average diameter of 694 ± 28.00 nm. A large nozzle inner diameter produces coarser initial jet streams, requiring greater tensile force to form morphologically uniform fibers. Under fixed air pressure, the resulting fibers exhibited larger diameters. Using a needle with an inner diameter of 0.31 mm, the average fiber diameter is 618 ± 24.36 nm. The average fiber diameter has decreased, but the fibers remain adhered to one another. When the needle diameter decreases to 0.26 mm, the fibers display a smooth surface without structural defects, with an average fiber diameter of 526 ± 1.01 nm. Fibers prepared with a nozzle inner diameter of 0.24 mm exhibit an average diameter of 517 ± 8.83 nm. However, the excessively small needle diameter increased solution flow resistance, causing frequent needle clogging during spinning and resulting in beads.

### 3.4. Effect of Solution Concentration on Fiber Morphology

**Figure 5:**
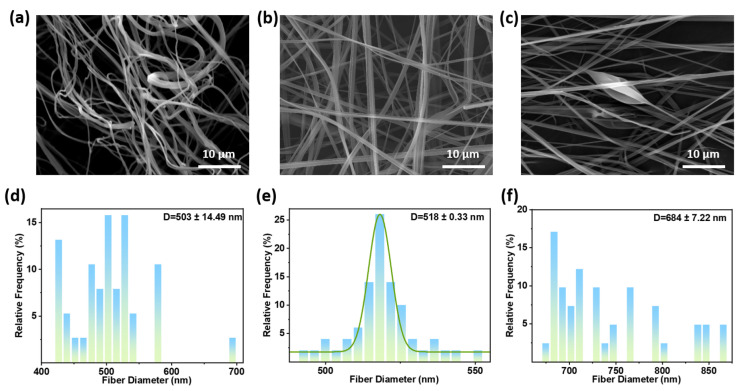
SEM images of PVB nanofiber prepared at solution concentrations of (**a**) 2 wt.%; (**b**) 5 wt.%; and (**c**) 8 wt.%. Fiber diameter distribution of PVB nanofiber prepared at solution concentrations of (**d**) 2 wt.%; (**e**) 5 wt.%; and (**f**) 8 wt.%.

Solution concentration is a fundamental parameter determining viscosity, surface tension, and chain entanglement density [37]. The viscosities of at 2 wt.%, 5 wt.%, and 8 wt.% PVB spinning solutions are 1.45, 40.42, and 98.87 mPa·s, respectively. Figure 5 shows SEM images and fiber diameter distributions of fiber membranes prepared with different solution concentrations (air pressure 0.15 MPa, solution flow rate 0.15 mL/min, needle inner diameter 0.26 mm, collector distance 15 cm, room temperature). At low concentration of 2 wt.%, the average fiber diameter is 503 ± 14.49 nm. Insufficient polymer chain entanglement and excessively low solution viscosity caused capillary breakage of the jet during pneumatic stretching, preventing the formation of fibers with uniform diameter. When the solution concentration increases to 5 wt.% (Figure 5b), the average fiber diameter is 518 ± 0.33 nm. Polymer chain entanglement provided sufficient viscoelasticity to resist breakage, ultimately yielding smooth, bead-free fibers with uniform distribution. With a solution concentration of 8 wt.%, the average fiber diameter is 684 ± 7.22 nm. Excessive solution viscosity hindered the effective stretching of the ejected droplet by the airflow, resulting in increased fiber diameter and beads.

SEM images and diameter distributions of fiber membranes prepared under different air pressures, solution flow rates, needle inner diameters, and solution concentrations were investigated. The optimal process parameters for preparing continuous, uniformly distributed, and bead-free PVB fiber membranes with solution blow spinning were air pressure 0.15 MPa, solution flow rate 0.15 mL/h, needle inner diameter 0.26 mm, and solution concentration 5 wt.%.

### 3.5. Effect of Spinning Time on the Weight and Thickness

**Figure 6:**
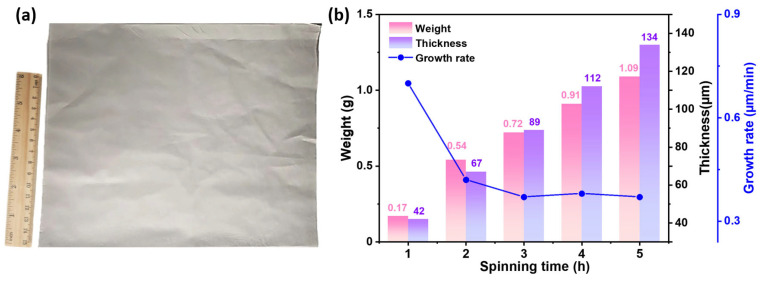
(**a**) Photo of the PVB fiber membrane prepared by solution blow spinning for 3 h; (**b**) weight, thickness, and growth rate of the PVB fiber membrane for different spinning times.

In addition to the fiber morphology, average fiber diameter and diameter uniformity, the growth efficiency of fiber membranes is also an important indicator for evaluating the preparation. The weight, thickness, and growth rate of fiber membranes prepared with different spinning times (1 to 5 h) are shown in Figure 6. As spinning time increases, the weight and thickness of a fibrous membrane progressively grows, while its growth rate declines. As spinning time increases, the thickness of the fiber membrane gradually increases. The newly prepared fibers were deposited onto the surface of fibers that had not fully dried. Residual solvents cause the fibers to stick together or partially fuse. The resulting densified structure impedes subsequent air flow and solvent evaporation, affecting subsequent fiber deposition. The solution blow spinning process employed in this study demonstrated significantly higher spinning efficiency compared to electrospinning. The thickness of fiber membranes obtained after 2 h of solution blow spinning was comparable to that achieved after 10 h of electrospinning [38]. Although existing research has rarely focused on membrane thickness, this parameter may influence the membrane’s porosity, barrier properties, mechanical characteristics, and active substance loading capacity. The thickness of fiber membranes used as antibacterial substrates typically ranges from 20 to 102 µm [39,40,41]. Therefore, in the subsequent study of this research, a PVB fiber membrane with a spinning time of 4 h was selected as a substrate. The thickness of fiber membranes achieved in this study ensures sufficient mechanical integrity for handheld operation, avoids brittleness issues associated with excessively thin membranes, and provides an ideal three-dimensional structural foundation for subsequent antibacterial agent loading. Further research is needed to enhance production efficiency and produce fibers with diameters, weights, and thicknesses that meet experimental requirements in less preparation time.

### 3.6. Morphology of ZnO NPs

**Figure 7:**
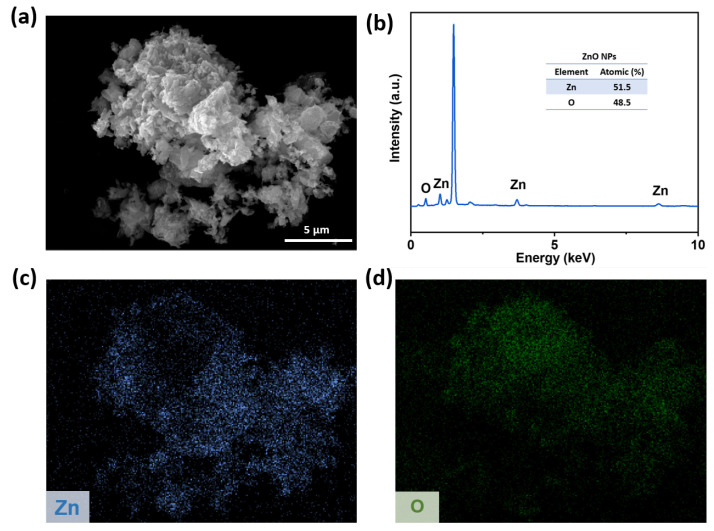
(**a**) Morphology of ZnO NPs; (**b**) EDX spectrum of ZnO NPs; and (**c**) Zn and (**d**) O elemental mapping images.

EDS was employed to characterize the chemical composition and elemental ratios of the ZnO NPs. Figure 7 presents the SEM, EDX, and elemental mapping images of the ZnO NPs. Figure 7a confirms that the commercially available ZnO NPs used in this study possess nanoscale dimensions and morphology. Figure 7b clearly displays characteristic peaks for zinc (Zn) and oxygen (O) elements. Quantitative elemental analysis results (Figure 7c,d) indicate atomic percentages of 51.5% for Zn and 48.5% for O.

### 3.7. FT-IR Spectroscopy

**Figure 8:**
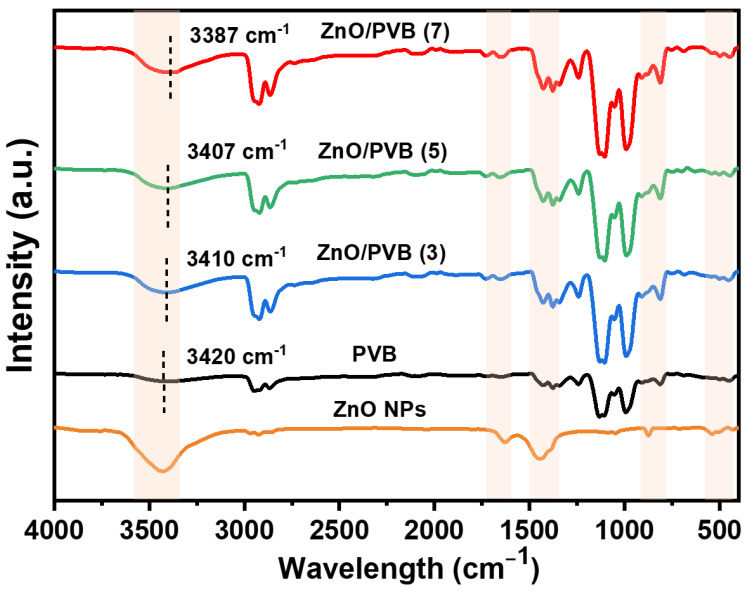
FT-IR spectra of ZnO NPs and PVB fiber membranes.

Figure 8 shows the FT-IR spectra of ZnO NPs and PVB fiber membranes. The characteristic absorption peaks of the PVB fiber membrane primarily include O–H stretching vibration peaks at 3330–3600 cm^−1^, saturated CH/CH_2_/CH_3_ group stretching vibrations at 2854 cm^−1^ and 2936 cm^−1^, C=O stretching vibrations at 1124 cm^−1^, and CO–C stretching vibration peaks at 990 cm^−1^ and 1130 cm^−1^. The Zn–O stretching vibration peak at 540 cm^−1^ and the hydroxyl absorption peak at 3429 cm^−1^ appear in the FT-IR of the ZnO/PVB fiber membranes; this confirms the successful incorporation of ZnO NPs into the PVB fiber membrane. As the mass ratio of the ZnO NPs gradually increased, the intensity of the O-H vibrational absorption band at 3200–3600 cm^−1^ in the ZnO/PVB fiber membrane was continuously enhanced [42]. Compared to the characteristic peaks of the PVB fiber membrane, the O-H absorption peak in the PVB/ZnO fiber membrane shifted from 3420 cm^−1^ to 3387 cm^−1^. This slight shift indicates the formation of hydrogen bonds between the ZnO NPs and polymer chains [43,44]. This may result from reactions between the PVB hydroxyl groups and ZnO NP hydroxyl groups during the drying process. Hydrogen bonding may improve the mechanical properties of fiber membranes [45,46].

### 3.8. Morphology and WCA of ZnO/PVB Fiber Membranes

**Figure 9:**
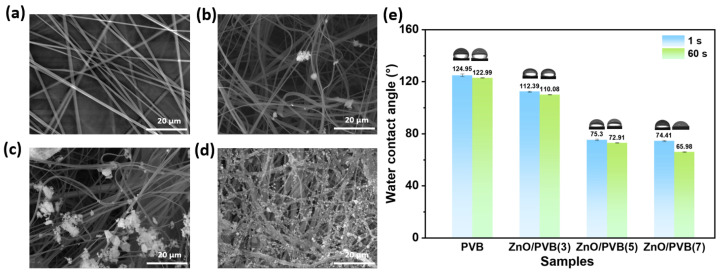
SEM images of (**a**) PVB; (**b**) ZnO/PVB(3); (**c**) ZnO/PVB(5); (**d**) ZnO/PVB(7); and (**e**) Photographs and water contact angle changes of all samples.

Figure 9 shows the SEM images and water contact angle measurements for PVB and ZnO/PVB fiber membranes. ZnO NPs were firmly anchored onto the surface of the fiber membranes, effectively infiltrating the pores of the fiber membranes. With increasing mass ratio of ZnO NPs, the fiber surface maintains smooth and morphologically uniform characteristics, indicating that the incorporation of ZnO NPs does not compromise the fibrous structure. Although the ZnO NPs were dispersed by ultrasonication, agglomeration still remains. The PVB fiber membrane exhibits strong hydrophobicity with a WCA of 125°. After incorporating ZnO NPs, the hydrophilicity of the fibrous membranes progressively increased. When the mass ratio of ZnO NPs is 3%, 5%, and 7%, the contact angles decreased to 112°, 75°, and 74°, respectively. The enhanced hydrophilicity of the ZnO/PVB fiber membranes is attributed to the surface hydroxyl groups of ZnO NPs, which lead to a corresponding increase in hydrophilicity as the nanoparticle mass ratio increases. To prevent internal moisture loss in fruits, packaging films typically require a certain degree of hydrophobicity. Therefore, the ZnO/PVB(3) fiber membrane with a WCA of 112° is considered more suitable for food packaging applications where moisture retention is critical [47,48].

### 3.9. Thermal Stability

**Figure 10:**
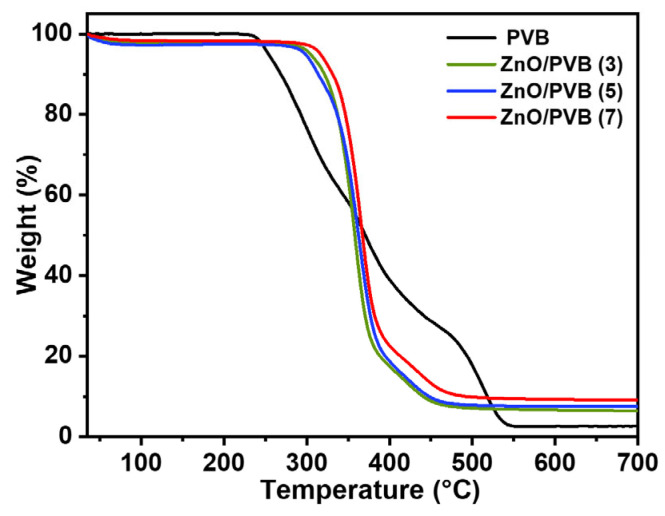
TG curves of PVB and ZnO/PVB fibrous membranes.

Figure 10 shows the TG curves of the PVB fiber membranes. Weight loss in the first stage (40–253.85 °C) primarily stems from the evaporation of residual moisture and physically adsorbed water within the sample. Weight loss in the second stage (220–450 °C) is mainly attributed to the decomposition of the polymer backbone, corresponding to PVB molecular chain breakage and pyrolysis processes. Compared to the PVB fiber membrane, the decomposition curves of the ZnO/PVB fiber membranes exhibit a shift toward higher temperatures in the elevated temperature region. This indicates that the incorporation of ZnO NPs significantly increases the thermal decomposition temperature of the PVB fiber membrane [33]. When temperature exceeds 450 °C, weight loss in the third stage is primarily driven by polymer carbonization and the stabilization of inorganic nanoparticles within the high-temperature zone. At this point, the decomposition rate of residual substances slows dramatically, causing the curve to flatten. Residues at 700 °C consist mainly of thermally stable ZnO NPs and trace carbonaceous residues that may form at elevated temperatures. Table 1 shows the T_5%_ of fiber membranes. As the mass ratio of ZnO NPs increases, the T_5%_ of the ZnO/PVB fiber membranes gradually increases while the weight loss rate gradually decreases, which indicates that ZnO NPs enhance the thermal stability of the fiber membranes. Compared to the T_5%_ of the PVB fiber membrane (253.85 °C), the T_5%_ of ZnO/PVB(7) increases to 316.25 °C. Excellent thermal stability is one of the fundamental properties enabling food packaging films to function effectively. It not only provides a broader processing window for food packaging production, allowing the film to withstand thermal stresses from processes like heat sealing and high-temperature sterilization, but also ensures the packaging maintains integrity and does not release harmful substances when exposed to unexpected high temperatures during storage and transportation. This stable heat resistance is crucial for extending food shelf life [49].

### 3.10. UV–Visible Spectroscopy

**Figure 11:**
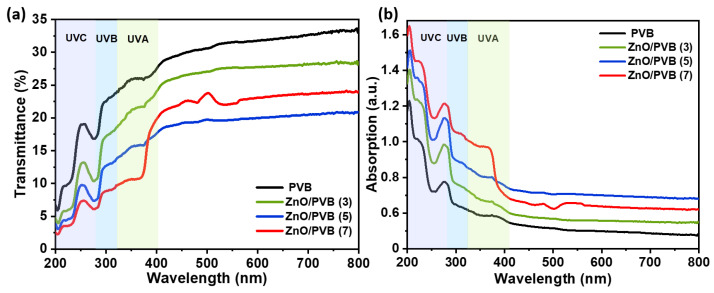
(**a**) UV transmittance spectra; (**b**) UV absorption spectra of PVB and ZnO/PVB fiber membranes.

The photon energy of ultraviolet radiation is sufficient to induce the breakage of multiple chemical bonds, posing a significant threat to polymers, food, and pharmaceuticals, and manifesting a decrease in material gloss and flexibility [50]. Consequently, developing effective UV protection measures holds significant importance across multiple fields. Figure 11 shows the ultraviolet-visible transmittance and absorption spectra of PVB fiber membranes.

The PVB fiber membrane exhibits moderate UV absorption properties, with UV transmittance below 28.73%. Incorporating ZnO NPs significantly enhances the UV absorption of the PVB fiber membrane. Within the 200 nm–400 nm wavelength range, the UV transmittance of ZnO/PVB fiber membranes decreases, reaching a minimum of 18.43%, demonstrating exceptional UV absorption capability. In the same range, the ZnO/PVB fiber membranes exhibit intense light absorption behavior, with absorption peak intensities significantly higher than those of the PVB fiber membrane. This enhancement is primarily attributed to the unique UV absorption characteristics of ZnO as a wide-bandgap semiconductor. As the mass ratio of ZnO NPs increases, the absorption intensity progressively enhances, confirming that ZnO NPs are the key component to conferring ultraviolet protection properties. In the visible region (460 nm–570 nm) of the UV-Vis spectrum for ZnO/PVB(7), new transmission valleys and absorption peaks emerge, indicating the formation of new chromophores within the ZnO/PVB system. This phenomenon likely stems from two factors. Firstly, oxygen atoms from hydroxyl and acetyl groups on PVB polymer chains form stable coordination bonds with Zn^2+^ ions on the ZnO surface, acting as efficient charge-transfer bridges to enhance interfacial charge-transfer transitions. Secondly, as the mass fraction of ZnO NPs increases, the hydrogen-bond network between PVB and ZnO NPs strengthens. Long PVB polymer chains form a scaffolding on the ZnO NP surface with hydrogen bonding, effectively preventing oxygen vacancies from aggregating into defect clusters and contacting ambient oxygen. This enhances the stability and extends the persistence duration of oxygen vacancies [51].

The ZnO/PVB fiber membrane effectively absorbed ultraviolet light while maintaining high visible light transmittance. These characteristics make it an ideal candidate for advanced food packaging that must integrate effective UV barrier functions with a transparent appearance [7].

### 3.11. Tensile Strength

**Figure 12:**
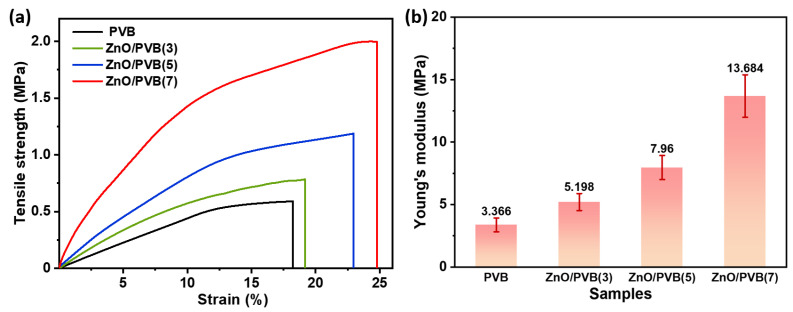
(**a**) Tensile strength and (**b**) Young’s modulus of PVB fiber membranes.

During transportation and distribution, packaging materials must possess the capacity to withstand heavy loads. Therefore, materials with excellent tensile strength are crucial in food packaging [52]. Figure 12 is the tensile stress–strain curves of the PVB fiber membranes. As the mass ratio of ZnO NPs increases, the tensile strength, tensile strain, and Young’s modulus of the fiber membranes progressively increase. The tensile strength of ZnO/PVB(7) reaches 1.995 MPa, representing a 238% improvement over the PVB fiber membrane (0.591 MPa). This enhancement primarily stems from hydrogen bonds formed between ZnO NPs and PVB polymer chains (Figure 8) [53]. The ZnO/PVB fiber membranes also exhibit a high modulus of up to 16.62 MPa, which prevents packaging from collapsing during transportation and shelf storage, thereby protecting internal food from crushing [54,55]. Despite their lower tensile strength and elongation than electrospun fiber membranes, solution blow spun PVB fiber membranes provide adequate mechanical strength for food packaging application, maintaining structural integrity under handling stresses during subsequent antibacterial testing [56].

### 3.12. Antibacterial Performance

**Figure 13:**
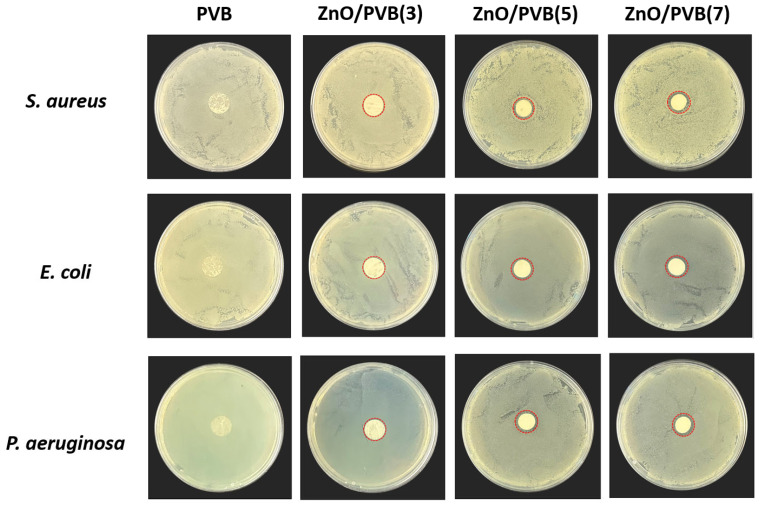
Antibacterial zones of PVB fiber membranes against *S. aureus*, *E. coli*, and *P. aeruginosa*.

This study employed the Kirby–Bauer test to evaluate the antibacterial performance of fiber membranes under conditions closer to real-world applications. Figure 13 shows photos of the antibacterial zone tests for PVB fiber membranes. The PVB fiber membranes exhibited no antibacterial properties. When doped with 3 wt.% to 7 wt.% ZnO NPs, all fiber membranes formed antibacterial zones. Table 2 shows the inhibition zone diameters of the fiber membrane against *S. aureus*, *Escherichia coli*, and *P. aeruginosa*. As the mass ratio of ZnO NPs increased, the diameter of the inhibition zone gradually expanded. ZnO/PVB fiber membranes exhibited antibacterial activity against *S. aureus*, *Escherichia coli*, and *P. aeruginosa*, with inhibition zone diameters of 10.77 ± 0.13 mm against *S. aureus*, 10.59 ± 0.17 mm against *E. coli*, and 10.62 ± 0.12 mm against *P. aeruginosa*. The antibacterial activity of fiber membranes in K−B tests was slightly lower than that reported in existing studies [57]. The antibacterial efficacy of ZnO/PVB fiber membranes requires further validation in practical applications.

**Figure 14:**
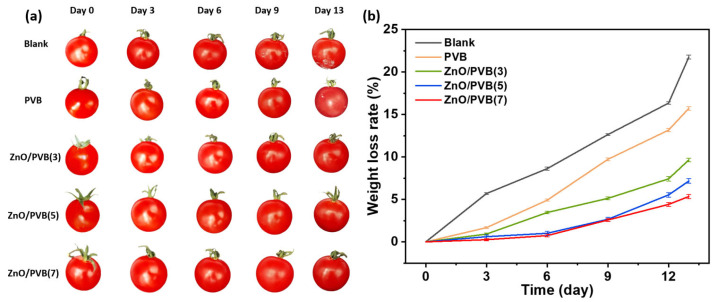
(**a**) Photos of cherry tomatoes at 13 days; (**b**) weight loss rate of cherry tomatoes.

To evaluate the effectiveness of PVB fiber membranes prepared with solution blow spinning in fruit preservation, cherry tomatoes were selected as the preservation test sample. Figure 14 shows photographs of cherry tomatoes and their mass loss rates during the 13 day storage period. Under conditions of 25 °C temperature and 50% relative humidity, unpackaged tomatoes (blank control group) were compared with tomatoes packaged using PVB and ZnO/PVB fiber membranes. Neither unpackaged nor packaged tomatoes exhibited signs of spoilage or wilting until day 12. On day 13, the control group exhibited significant water loss and spoilage, with extensive white mold spots appearing on the surface. Tomatoes packaged with PVB fiber membranes showed scattered mold spots, indicating an early stage of mold growth. The PVB fiber membrane lacks antibacterial properties; while it blocks some external bacteria and light radiation, it cannot release antibacterial agents to actively inhibit microbial growth. However, tomatoes packaged with ZnO/PVB fiber membranes remained intact until day 13. While their color vibrancy, gloss, and overall quality gradually diminished over time, with stem ends yellowing and dehydration occurring, no signs of decay were observed. This may be attributed to the fiber membrane blocking most ultraviolet and visible light, thereby reducing light exposure. Simultaneously, the fiber membrane blocked most bacteria and dust, thereby slowing the rate of tomato dehydration and decay. Tomatoes gradually lost water over time, with the control group experiencing the highest dehydration rate of 21.75% weight loss until day 13. Tomatoes packaged with PVB fiber membranes exhibited significantly reduced weight loss compared with the control group. As the ZnO NPs increased, the weight loss of tomatoes packaged with ZnO/PVB fiber membranes gradually decreased. Tomatoes packaged with ZnO/PVB(7) achieved a low weight loss of 5.34% over 13 days. Therefore, the use of ZnO/PVB fiber membranes effectively reduced tomato moisture loss, blocked external bacteria and dust, and extended the shelf life to 13 days, surpassing 7-day shelf life extension achieved in similar study [58].

## 4. Conclusions

In this study, by optimizing the key process parameters of solution blow spinning, the optimal preparation conditions were an air pressure of 0.15 MPa, a solution flow rate of 0.15 mL/h, a needle inner diameter of 0.26 mm, and a solution concentration of 5 wt.%. ZnO NPs synergistically enhanced the UV absorption, thermal stability, mechanical strength, and antibacterial activity of PVB nanofiber membranes. Cherry tomatoes packaged with ZnO/PVB(7) nanofiber membranes exhibited a mass loss rate of only 5.34% over 13 days, significantly lower than the 21.75% loss in the untreated control group, extending tomato shelf life to 13 days. This study provides a feasible pathway for the efficient, low-cost utilization of waste PVB. The developed ZnO/PVB composite fiber membrane integrates UV absorption, thermal stability, mechanical reinforcement, and antibacterial functionality, demonstrating promising application prospects in the field of active food packaging.

## Figures and Tables

**Table 1:** T_5%_ of fiber membranes.

Samples	T_5%_
PVB	253.85
ZnO/PVB(3)	298.51
ZnO/PVB(5)	304.67
ZnO/PVB(7)	316.25

**Table 2:** Antibacterial zone diameter of the fiber membrane.

Samples	*S. aureus*	*E. coli*	*P. aeruginosa*
PVB	—	—	—
ZnO/PVB(3)	10.41 ± 0.31	9.6 ± 0.82	10.21 ± 0.03
ZnO/PVB(5)	10.37 ± 0.16	10.55 ± 0.11	10.71 ± 0.62
ZnO/PVB(7)	10.77 ± 0.13	10.59 ± 0.17	10.62 ± 0.12

## Data Availability

The data that support the findings of this study are contained within the article. Further inquiries can be directed to the corresponding authors.
